# Robust Feedback Zoom Tracking for Digital Video Surveillance

**DOI:** 10.3390/s120608073

**Published:** 2012-06-11

**Authors:** Tengyue Zou, Xiaoqi Tang, Bao Song, Jin Wang, Jihong Chen

**Affiliations:** National NC System Engineering Research Center, Huazhong University of Science and Technology, Wuhan 430074, China; E-Mails: zouty@smail.hust.edu.cn (T.Z.); xqtang@hust.edu.cn (X.T.); iamlaoking@smail.hust.edu.cn (J.W.); jhongchen65@gmail.com (J.C.)

**Keywords:** zoom tracking, video surveillance, focus value, focus control, PID feedback

## Abstract

Zoom tracking is an important function in video surveillance, particularly in traffic management and security monitoring. It involves keeping an object of interest in focus during the zoom operation. Zoom tracking is typically achieved by moving the zoom and focus motors in lenses following the so-called “trace curve”, which shows the in-focus motor positions versus the zoom motor positions for a specific object distance. The main task of a zoom tracking approach is to accurately estimate the trace curve for the specified object. Because a proportional integral derivative (PID) controller has historically been considered to be the best controller in the absence of knowledge of the underlying process and its high-quality performance in motor control, in this paper, we propose a novel feedback zoom tracking (FZT) approach based on the geometric trace curve estimation and PID feedback controller. The performance of this approach is compared with existing zoom tracking methods in digital video surveillance. The real-time implementation results obtained on an actual digital video platform indicate that the developed FZT approach not only solves the traditional one-to-many mapping problem without pre-training but also improves the robustness for tracking moving or switching objects which is the key challenge in video surveillance.

## 1. Introduction

**Figure 1.**
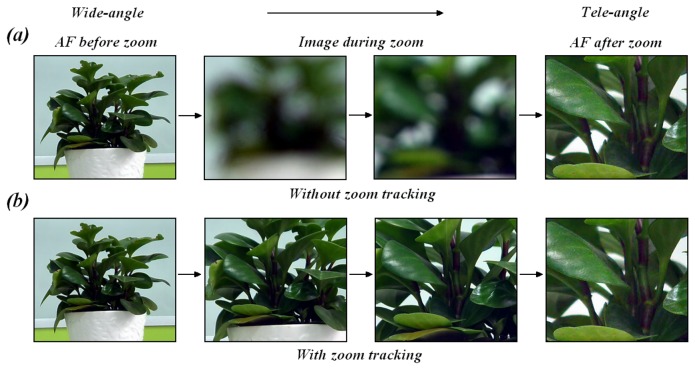
Illustration of the zoom tracking effect.

Due to the remarkable growth in the video surveillance market over the last few years [1–3], high-quality imaging results from zoom operation are now demanded by consumers [4,5], particularly in traffic management and security monitoring [6–8]. Maintaining image sharpness or focus during the entire zoom process is the main challenge of zoom tracking. Figure 1 shows the zoom tracking effect as the zoom is changed from a wide-angle zoom to a tele-angle zoom. As shown in this figure, the plant remains in-focus as the zoom is changed by the user in the presence of zoom tracking. However, the image becomes out-of-focus in the absence of zoom tracking, and the image finally clarifies after zoom tracking due to an auto-focusing (AF) [9] algorithm.

### 1.1. Zoom Tracking Principle

**Figure 2.**
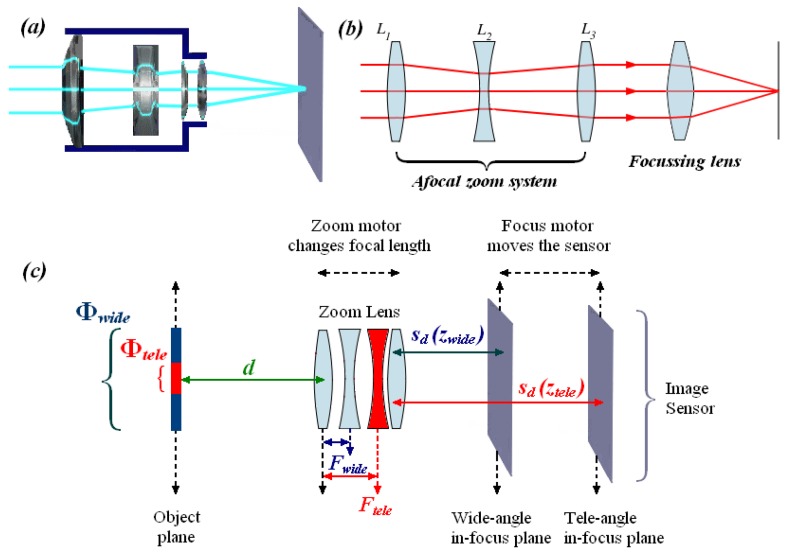
(**a**) Illustration of an actual zoom system; (**b**) The structure of a zoom system; (**c**) Zoom tracking mechanism.

Users often utilise two different zoom options in a digital video system: optical zoom and digital zoom. Digital zoom works by cropping and subsequently enlarging a captured image, which produces an image of lower optical resolution. In contrast, optical zoom uses the optic lens to bring the subject closer [10]. In this paper the zoom tracking problem is only studied for optical zoom. Figure 2(a) shows an actual zoom system, and its structure chart is shown in Figure 2(b).

**Figure 3.**
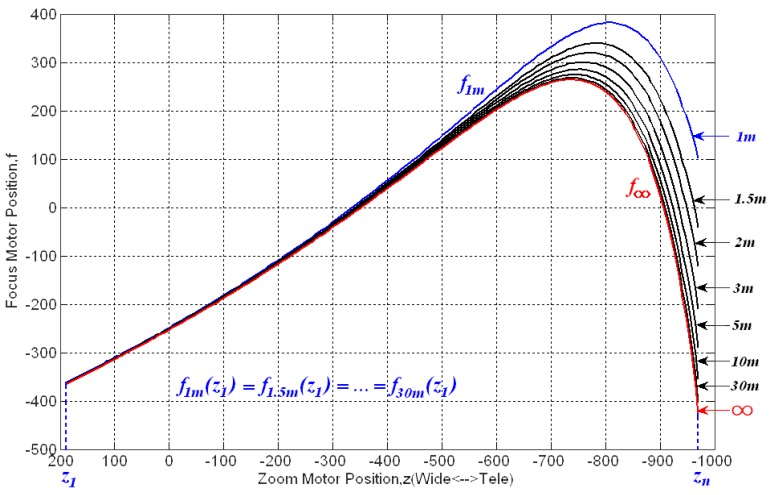
Trace curves for an 18× zoom lens.

Figure 2(c) introduces the zoom tracking mechanism in detail. When the zoom is changed from wide-angle to tele-angle, the zoom lens focal length increases from *F*_wide_ to *F*_tele_, whereas the angle of view reduces from *Φ*_wide_ to *Φ*_tele_. In response to this change, the in-focus plane (image distance) should shift during this process. For an object at a distance *d*, *s_d_(z_wide_)* and *s_d_(z_tele_)* are defined as the image distance at wide-angle and tele-angle zooms, respectively. Thus, when the zoom is changed from wide-angle to tele-angle, to maintain image sharpness, the image sensor must be moved from the wide-angle in-focus plane at *s_d_(z_wide_)* to the tele-angle in-focus plane at *s_d_(z_tele_)*. As the zoom lens focal length is altered via a zoom motor and the image sensor is moved by a focus motor, the zoom tracking is typically achieved by following the so-called “trace curves”, which show zoom motor positions *versus* in-focus motor positions for various object distances in Figure 3. Thus, trace curve estimation is a crucial problem for zoom tracking methods. A major challenge in this estimation is the one-to-many mapping problem [11], which becomes troublesome when the zoom is changed from wide-angle to tele-angle. This problem will be further described in Section 2.

### 1.2. Existing Zoom Tracking Methods

The existing zoom tracking methods can be divided into two categories: (1) geometric methods, such as geometric zoom tracking (GZT) and adaptive zoom tracking (AZT); (2) machine learning methods, such as relational zoom tracking (RZT) and predictive zoom tracking (PZT). The development of zoom tracking can be traced back to the look-up table method [12], which stores a large number of trace curves for various object distances in memory. The real trace curve is estimated by selecting the closest curve among the stored ones. However, this approach is not often used in practice because of its large memory requirement. To cover the shortage of memory, the GZT [13,14] has been proposed. The GZT approach obtains an estimate of a trace curve via linear interpolation only based on two trace curves for near and far objects. A drawback of this approach is that the offset between the estimated and the real trace curves gradually increases as the zoom is changed from wide-angle to tele-angle. This approach is later extended to the AZT method [15], which incorporates a recalibration procedure at the boundary zoom position where the trace curve changes from linear to non-linear.

The RZT [16] and PZT [11] methods were proposed later to improve the estimation accuracy through machine learning. RZT generates an estimate of the distance range in which the object resides by so-called “relational curves”. This distance range is then used to estimate a trace curve. PZT uses an input-output model trained by *a priori* characteristic trace curves to generate an estimate of a trace curve. The trained model is often based on the Auto-Regression with Exogenous Inputs (ARX) model [17] or the Recurrent Neural Network (RNN) model [18]. Both RZT and PZT solve the one-to-many mapping problem well, but they require a significant amount of *a priori* knowledge for training. It is not always convenient to obtain these *a priori* trace curves in practical use. Furthermore, the errors in the learning step will also have an effect on the estimation. Because the variation of the lens or scenes often requires additional time for re-training, the adaptability of these two algorithms is relatively poor.

### 1.3. Zoom Tracking for Digital Video Surveillance

There are typically two occasions for which the optical zoom is used: (1) the enlarged occasion, which enlarges the object at a constant distance in image to look at it in detail; and (2) the telephoto occasion, which tracks the object moving away. In traffic management and security monitoring, the telephoto occasions are often encountered, for example, for capturing the license plate of an escaping vehicle that has just run a red light. However, all existing zoom tracking methods mentioned previously have been developed for the digital still camera systems. These algorithmic methods assume that the object distance is constant; thus, the moving or switching object in video surveillance [19] has not been considered.

**Figure 4.**
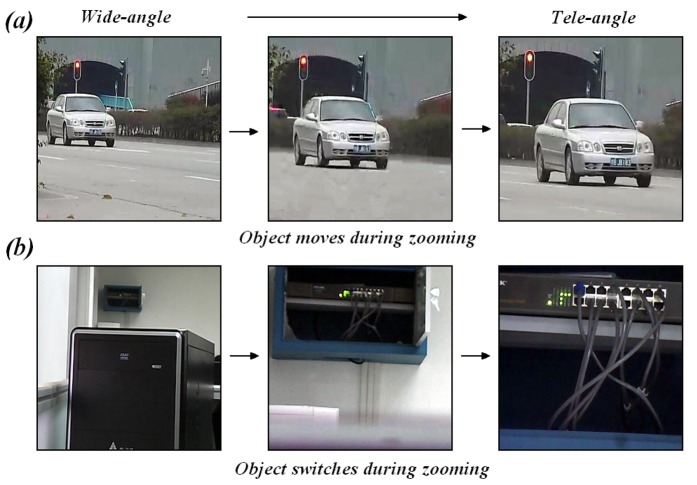
(**a**) Moving object during zooming; (**b**) Switching object during zooming.

Figure 4(a) shows the moving object as the zoom is changed from wide-angle to tele-angle. The object distance is changing as the car moves towards the video camera during zooming. In this situation, existing methods cannot produce an ideal result. There are several other situations in which these methods cannot properly function, even when the objects are stationary. Figure 4(b) illustrates the switching object during zooming. The computer box and network switch are shown as two stationary objects at different distances in the scene. When the zoom motor is moved from wide-angle to tele-angle, the main target in the video changes from the computer box to the network switch. The traditional zoom tracking methods will also fail in this situation.

To track moving and switching objects in digital video surveillance and to acquire better estimated results without pre-training the system, we propose the robust feedback zoom tracking (FZT) method to revise the estimated trace curve, which is based on traditional GZT estimation and utilises a proportional-integral-derivative (PID) loop-closed feedback controller [20–22]. In the absence of knowledge of the underlying process, a PID controller has historically been considered optimal [23]. The controller can provide control action for specific process requirements by tuning its parameters. This method compensates for errors along the estimated trace curve using the real-time focus value (FV), which is typically used in the auto-focusing function.

### 1.4. Contributions and Organisation

In this work: (1) we discuss the zoom tracking methods in video surveillance for the first time; (2) we propose a novel zoom tracking method called FZT, which is robust in tracking moving or switching objects in video surveillance; (3) we implement our FZT zoom tracking algorithm on real-time digital video hardware and compare it with commonly used algorithms. To the best of our knowledge, the focus value and real-time feedback mechanism have not yet been used in previous zoom tracking studies, and there have been no previous reports on the implementation of the zoom tracking method in video surveillance devices.

This paper is organised as follows. Section 2 introduces our FZT method in detail. The FZT approach is then implemented on the hardware platform in Section 3. Our experimental results and comparisons between our algorithm and other existing methods in terms of accuracy and speed are reported in Section 4. Finally, conclusions are stated in Section 5.

## 2. Feedback Zoom Tracking

As mentioned above, zoom tracking is related to the zoom and focus motor positions. It is typically achieved by following a trace curve. If the motors are moved following the trace curve during zoom operation, the image will always stay sharp. Figure 3 shows the trace curves for an 18× zoom lens. Each trace curve corresponds to a certain object distance.

### 2.1. Trace Curve Estimation

The first goal in zoom tracking that we addressed is how to estimate the right trace curve without any special distance measurement equipment. Let *f*_d_ denote the real trace curve acquired by running the global search auto-focusing function [24,25]. Thus, *f*_d_ indicates the in-focus motor position for each zoom motor position *z_n_* at a given object distance *d*. For simplicity, let *z_1_* and *z_n_* denote the wide-angle zoom (*z_wide_*) and tele-angle zoom (*z_tele_*), respectively. As shown in Figure 3, all of the trace curves for various object distances have the same in-focus motor position at the wide-angle zoom *z_1_*, which is *f*_1m_ (*z_1_*) = *f*_1.5m_ (*z_1_*) =… = *f*_30m_ (*z_1_*). However, it is difficult to determine which trace curve should be followed during zooming without the distance information, particularly when the zoom motor moves from the wide-angle towards the tele-angle. This issue is the so-called “one-to-many” mapping problem.

**Figure 5.**
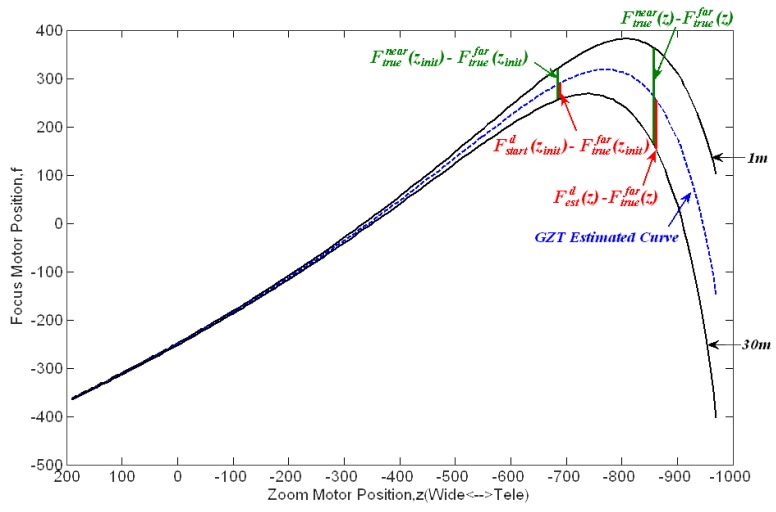
Illustration of the GZT focus ratio.

Thus, a zoom tracking approach is required to estimate a trace curve as close as possible to the real one. The classical method GZT estimates the trace curve via linear interpolation based on the stored trace curves for near and far objects. It obtains the estimated trace curve using Equation (1):

(1) $$F_{\text{est}}^{d}(z)=\{\begin{array}{ll} F_{\text{start}}^{d}(z_{\text{init}}) & z=z_{\text{init}}\\ \left(\frac{F_{\text{start}}^{d}(z_{\text{init}})-F_{\text{true}}^{\text{far}}(z_{\text{init}})}{F_{\text{true}}^{\text{near}}(z_{\text{init}})-F_{\text{true}}^{\text{far}}(z_{\text{init}})}\right)*\left(F_{\text{true}}^{\text{near}}(z)-F_{\text{true}}^{\text{far}}(z)\right)+F_{\text{true}}^{\text{far}}(z) & z\neq z_{\text{init}} \end{array}$$

where $F_{\text{true}}^{\text{near}}(z)$ and $F_{\text{true}}^{\text{far}}(z)$ denote in-focus motor positions at the zoom position *z* for near and far objects, respectively, and *z_init_* and $F_{\text{start}}^{d}(z_{\text{init})}$ represent the initial zoom motor position and its corresponding in-focus motor position for an object at a distance *d*, respectively. The subscript “*start*” indicates that the in-focus motor position is obtained by performing auto-focusing before the zoom motor is moved. As shown in Figure 5, GZT actually uses the so-called GZT focus ratio described in Equation (2) to estimate the in-focus motor position:

(2) $$\frac{F_{\text{start}}^{d}(z_{\text{init}})-F_{\text{true}}^{\text{far}}(z_{\text{init}})}{F_{\text{true}}^{\text{near}}(z_{\text{init}})-F_{\text{true}}^{\text{far}}(z_{\text{init}})}=\frac{F_{\text{est}}^{d}(z)-F_{\text{true}}^{\text{far}}(z)}{F_{\text{true}}^{\text{near}}(z)-F_{\text{true}}^{\text{far}}(z)}$$

**Figure 6.**
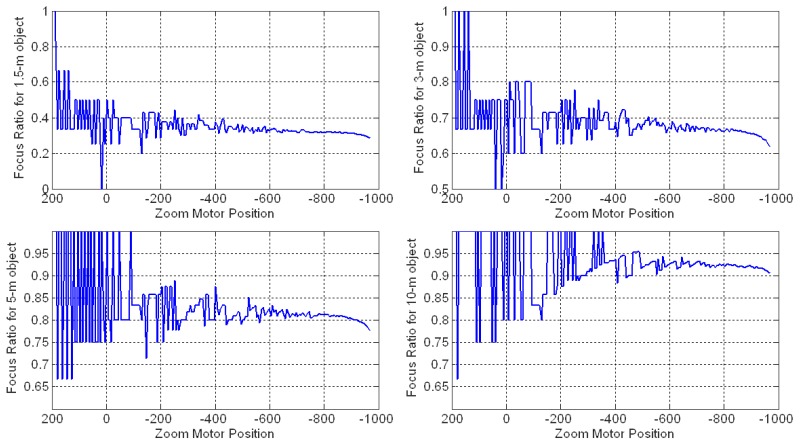
GZT focus ratio curves.

Figure 6 shows the effectiveness of the GZT focus ratio for the targets at different distances. When the zoom is changed from wide-angle to tele-angle, the GZT focus ratio shows non-linear characteristics, resulting in large estimation errors when predicting the trace curves with GZT. Although AZT uses recalibration to improve its accuracy, it also cannot completely avoid this type of error caused by using linear interpolation to fit the non-linear problem.

### 2.2. Trace Curve Revision

**Figure 7.**
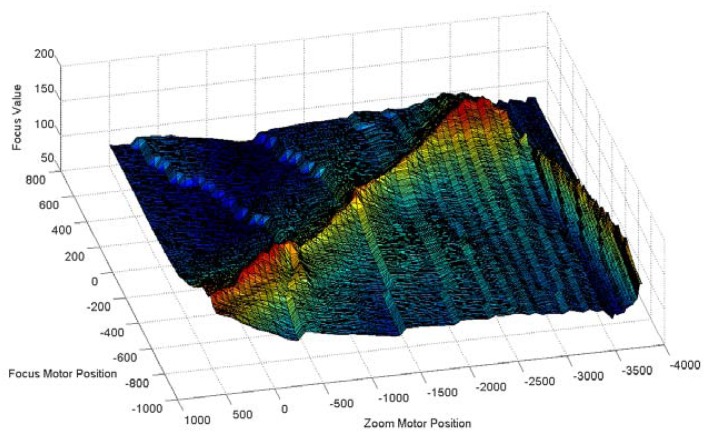
Focus values for each motor position.

To overcome the disadvantages of GZT and the issues associated with the moving and switching objects, we utilise the feedback method to revise the estimated trace curve automatically in real-time applications. The first step of the feedback method is to acquire the error from the system. We first consider the focus value (FV) [26–28], which is the measurement of sharpness in the auto-focusing application. As the focus value increases, an object's image increases in sharpness. Figure 7 illustrates the focus values for per focus motor position *versus* per zoom motor position acquired using our digital video surveillance equipment, which is described in Section 3. Figure 7 shows that the highest focus value is on the peak of the mountain and that sharpness decreases gradually down the hillside. The peak line is the real trace curve for the object in the experiment. Away from the trace curves, the corresponding focus value declines symmetrically on both sides of the mountain. Thus, the FV can be used as a measurement of the offset between a test point and the real trace curve.

**Figure 8.**
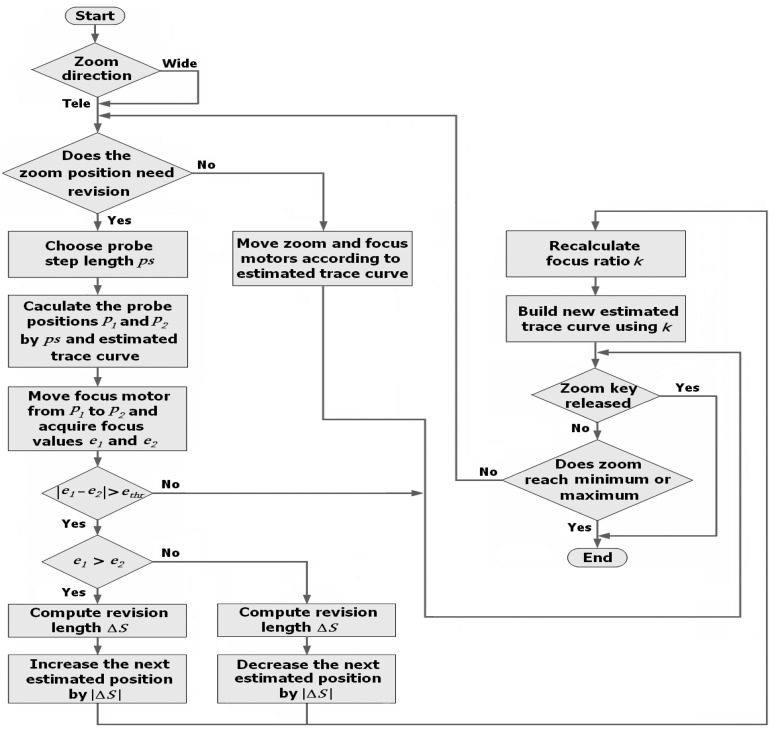
Feedback zoom tracking method.

Using focus values, we propose that the FZT method will maintain object sharpness during the entire zoom process, even when there are moving or switching objects in the scene. Figure 8 depicts the FZT method flowchart. There are three main stages in this flowchart: *detection position estimation*, *feedback value calculation*, and *trace curve revision*.

**Figure 9.**
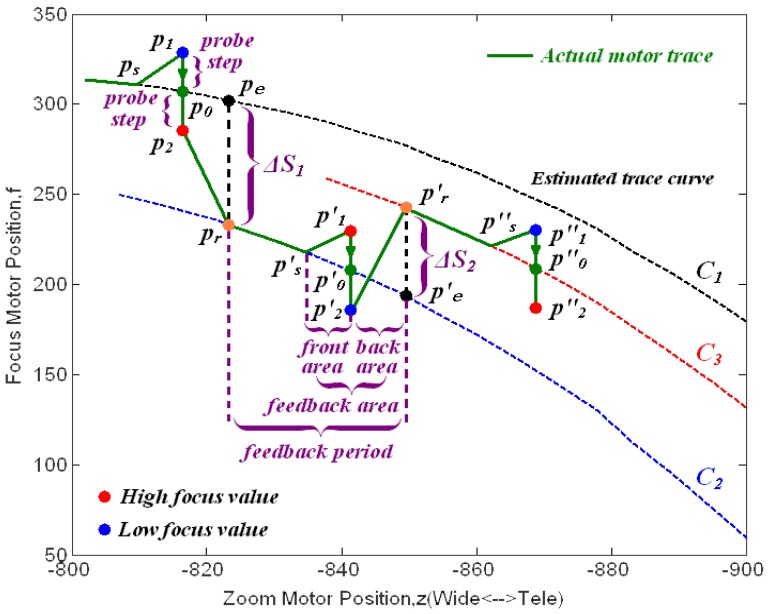
Illustration of FZT revision.

In the first stage, the initial estimated trace curve is given by the GZT model according to the geometric characteristics at the beginning of zooming. When the user changes the zoom from wide-angle to tele-angle, the approach requires a feedback period length *fp* to determine where it should revise the trace curve. If *fp* = 48, the system must detect the error once every 48 zoom motor steps. For example, if the first detection begins at motor position *z* = −2,536, the following feedback mechanism will be run at z = −2,584, −2,632, −2,680, and so on. As shown in Figure 8, if the current zoom position does not require revision, the zoom and focus motors are moved according to the current estimated trace curve without detection; otherwise, the system would acquire the focus values at two corresponding probe points for real-time feedback revision. The probe points are detecting positions for obtaining the focus values needed by our FZT, and they are symmetrically located on both sides of the current estimated trace curve. Figure 9 shows that the two probe point positions *p_1_* and *p_2_* are calculated using *p_1_* = *p_0_* + *ps*, *p_2_* = *p_0_− ps*, in which *p_0_* is the point on the current estimated trace curve at the corresponding zoom position, and *ps* is a probe step length parameter used to determine positions *p_1_* and *p_2_*. This *ps* parameter controls the detection boundary of the algorithm. A small *ps* may miss some tiny errors, whereas a large *ps* will increase the fluctuation of the trace curve. The *ps* can be either constant or variable. Here, we propose an adaptive selection mechanism: the *ps* is determined using the difference between the current and next focus motor positions on the estimated trace curve. The adaptive mechanism is described by Equation (3):

(3) $$\{\begin{array}{rr} ps=2 & ,0<F_{\text{next}}-F_{\text{current}}\leq 10\\ ps=4 & ,10<F_{\text{next}}-F_{\text{current}}\leq 20\\ ps=6 & ,20<F_{\text{next}}-F_{\text{current}}\leq 30\\ ps=8 & ,20<F_{\text{next}}-F_{\text{current}}\leq 40 \end{array}$$

where *F_current_* represents the focus motor position on the estimated trace curve at the current step, and *F_next_* represents the focus motor position at the next step. Both of these are shown on the y-axis in Figure 9.

**Figure 10.**
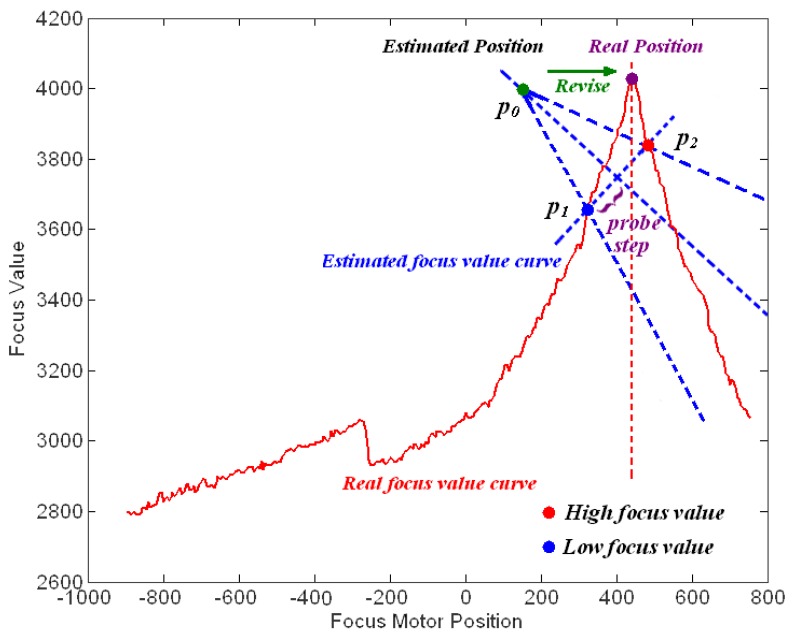
Principle of FZT revision.

In the second stage, the focus motor is moved from *p_1_* to *p_2_*, and the corresponding focus values *e_1_* and *e_2_* are acquired at these two points, respectively. Because the focus value decreases symmetrically on both sides with the increasing distance from the real trace curve, the revision can be made by our FZT algorithm. Because they are the same distance from the point *p_0_*, the probe points *p_1_* and *p_2_* should have approximately the same focus value. However, because the estimated trace curve often deviates from the real one, the focus values *e_1_* and *e_2_* are often different. By studying the relationship between these two values, we can determine the actual location of the trace curve. As illustrated in Figure 10, the red line represents the real focus value curve at the current zoom position, whereas the blue line represents the estimated focus value curve. Thus, *e_2_* > *e_1_* in Figure 10 indicates that the probe point *p_2_* is closer to the real trace curve than is *p_1_*. Thus, the estimated trace curve should be revised towards the direction of *p_2_* to approach the real one. In contrast, if *e_1_* > *e_2_*, the estimated trace curve should be moved towards *p_1_*.

During the *trace curve revision* stage, revision is achieved by moving the next estimated position *p_e_* on the current estimated trace curve to *p_r_*, as shown in Figure 9. The program then updates the GZT focus ratio *k* by *p_r_* and rebuilds the estimated trace curve. The revision distance Δ*S*, which will be discussed later, is finally calculated by the PID controller.

In addition to the feedback period *fp*, there are several other variable parameters in our FZT model. In the feedback area, the motors are moved following straight lines. The feedback area length *fa*, which consists of the front area length *fra* and the back area length *bka*, influences the fluctuations of the motor trace. A large *fa* value will reduce the slope of the trace adjustment and causes less shaking in the image during the process. In other parts of the feedback period, the motors should be moved according to the current estimated trace curve.

The process of revision is given in Figure 9. When the zoom motor enters the first feedback area at point *p_s_*, the probe position *p_1_*, *p_2_* should be calculated at the next step. The motors are then moved to these positions to acquire the focus values *e_1_* and *e_2_* following straight lines. The error can then be obtained using Equation (4):

(4) $$\Delta e=e_{1}-e_{2}$$

Because Δ*e* < 0 and |Δ*e*| > *e_thr_*, the estimated trace curve should be revised towards the *p_2_* direction in which *e_thr_* is a threshold parameter that avoids system jittering. Next, the position *p_e_* on the current estimated trace curve is revised to *p_r_* = *p_e_* + Δ*S_1_*. The focus ratio *k* is then recalculated via the position of *p_r_*, and the new estimated trace curve *C_2_* is built by the classical GZT method.

The motors pass through the back area following the straight line from *p_2_* to *p_r_*. They then move from *p_r_* to *p*′*_s_* following the curve *C_2_* without feedback and enter the second feedback area. Because Δ*e* > 0 in this area, the estimated trace curve *C_2_* is judged to have a lower value than the real trace curve. Then, the position *p*′*_e_* is revised to *p*′*_r_* on the curve *C_3_* by Δ*S_2_*. The green line in Figure 9 shows the actual motor trace during this process. The feedback mechanism occurs during the entire zoom operation process.

### 2.3. Revision Distance Control

**Figure 11.**
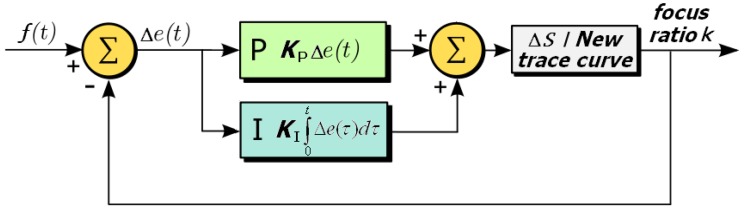
Control structure of FZT.

The revision distance Δ*S* is a critical parameter that decides the regulating ability of the algorithm. If the Δ*S* is smaller than the ideal Δ*S*, the revised trace curve will not approach the real trace curve efficiently. However, if Δ*S* is too large, an overshoot error will occur. Because the revision is influenced not only by the current error but also by the previous errors, we use a proportional-integral (PI) controller to improve its accuracy. The PI controller, which is widely used in motor control, can provide the control action according to the current and previous errors. Figure 11 shows the control structure of our FZT method. The controller action, which consists of proportional and integral components, can also be written as Equation (5):

(5) $$\Delta S=K_{P}\left[\Delta e(t)+\frac{1}{T_{I}}\int_{0}^{t}\Delta e(\tau )d\tau \right]$$

where *K_P_* is the proportional gain and *T_I_* is the integral time. The integral component accumulates all previous errors to compensate for the error value, with the intention of completely eliminating these errors in *T_I_* seconds. The resulting compensated error value is scaled by the proportional gain *K_P_*.

Because the Equation (5) can only be used in analogue systems, the integral component should be discretised for the digital equipment. Equation (6) shows the formula conversion from the integral term to the sum of discrete errors:

(6) $$\int_{0}^{t}\Delta e(\tau )d\tau =\sum_{j=0}^{n}\Delta e(j)\cdot \Delta t=T\sum_{j=0}^{n}\Delta e(j)$$

where Δ*t* = *T* represents the sampling period. In our experiments, the value of *T* is set to 1. Equation (5) can then be rewritten in discrete form as Equation (7):

(7) $$\Delta S(k)=K_{P}\left[\Delta e(k)+\frac{T}{T_{I}}\sum_{j=0}^{N}\Delta e(j)\right]$$

According to Equation (8), Equation (7) can be further converted to the incremental form as Equation (9), which simplifies the calculation and saves storage space. This equation now only needs the last Δ*S* and the errors in the last two consecutive steps to calculate the revision distance:

(8) $$\Delta S(k)-\Delta S(k-1)=K_{P}\left[\Delta e(k)-\Delta e(k-1)+\frac{T}{T_{I}}\Delta e(k)\right]$$

(9) $$\Delta S(k)=\Delta S(k-1)+K_{P}[\Delta e(k)-\Delta e(k-1)]+K_{I}\Delta e(k)$$

where $K_{I}=K_{P}\frac{T}{T_{I}}$ is the integral coefficient.

Using the PI controller, FZT is able to complete its feedback procedure. However, the parameters *K_P_* and *T_I_* need tuning before use. Tuning a PI control loop involves adjusting these parameters to the optimum values for the desired control response. There are several methods for tuning a PI loop, including manual tuning, the Ziegler-Nichols method [29], the Cohen-Coon method [30] and so on.

## 3. Real-Time Hardware Implementation

**Figure 12.**
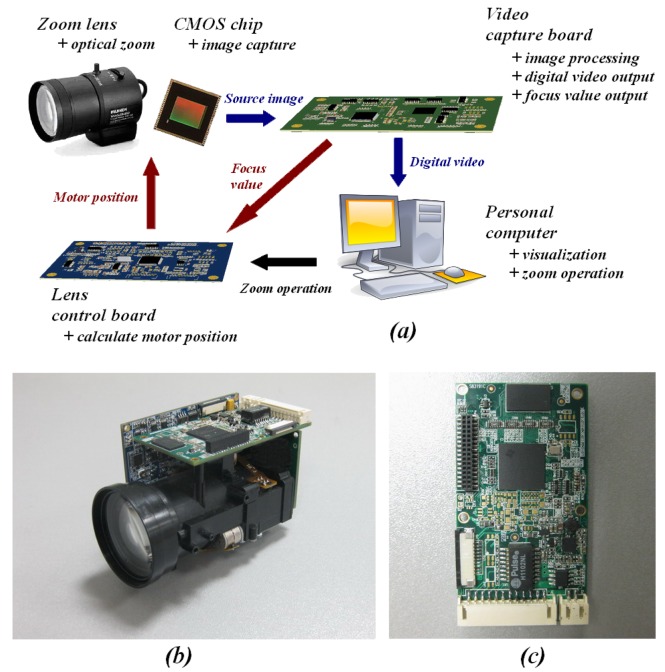
High-speed TI TMS320DM365 digital video platform.

The improved FZT algorithm and traditional methods were implemented on a high-speed TI TMS320DM365 digital video platform, and the focus value calculation for the 720-P (1280 × 720 pixels) image was simultaneously performed at 30f/s. Figure 12(a) shows the configuration of this platform. This platform consists of a zoom lens, CMOS chip, dedicated video capture board, lens control board, and PC.

For the high-speed camera head, we adopted a CHIOPT 18× zoom lens, in which the zoom range is sufficiently large for experiments. To increase motion accuracy, the zoom motor was driven by a program in four-subdivision mode, which divides each normal motor step into four smaller steps. Figure 12(b) shows an overview of the device. Twelve-bit RAW image data were built by the 5-MP CMOS chip (MT9P031) and then transferred to the video capture board at 30f/s for 1,280 × 720 pixels. The video capture board is designed as a dedicated device for video capturing, transferring, processing, and focus value calculation. Figure 12(c) shows an overview of this board, which consists of four parts: (1) an ARM microprocessor (TMS320DM365) for building video from CMOS and transferring it to a PC via Ethernet; (2) a C8051F microcontroller (C8051F360) for calculating the focus value and outputting it by RS485; (3) memories, including DDR-SDRAM (MT47H64M16HR); and (4) interface circuits, such as UART and Ethernet. This board, CMOS, and zoom lens actually construct a standard internet protocol network camera (IPNC) system.

The lens zoom board is another electronic function in this system. This board contains another C8051F microcontroller for estimating the trace curve. It receives zoom commands and focus values from the PC and video capture board, respectively. The FZT algorithm is implemented here to acquire the positions of motors using focus values. The motor control signals are then produced by the special motor control chip. The entire working procedure of our device is as follows:

1. *Receive zoom command from PC*: The PC transfers the zoom command given by the user to the lens control board.
2. *Acquire motor position by estimated trace curve:* Our FZT algorithm is an improved GZT that accounts for the focus value when revising the estimated trace curve. Before obtaining the corresponding focus value, the lens control board applies GZT to estimate the position of the focus motor. In the feedback area, the probe positions are also acquired on this board through FZT.
3. *Calculate the focus value:* The focus value is calculated by the video capture board and sent to the lens control board. To fit the real-time requirement, we use the analogue circuits. The corresponding analogue video signals are first output by the Video DAC in TMS320DM365. Then, an analogue band-pass circuit is used to filter out the high-frequency components. As the number of high-frequency components increases, the clarity of the image increases. A precise small-signal rectifier and analogue integrator circuit are applied to build a voltage from high-frequency components that represents the focus value. The 10-bit A/D converter embedded in the C8051F microcontroller is used to obtain the exact digital focus value from the voltage. Furthermore, the focus value can also be obtained digitally through the information contained in the H3A register in TMS320DM365.
4. *Revise the estimated trace curve using FZT:* The FZT is run on the lens control board to revise the estimated trace curve. The next motor positions can then be obtained by the new curve.
5. *Create control signals and move the motors:* The motor control signals are created by the special motor control chip in the lens control board according to the new trace curve. The zoom and focus motors are then moved to the exact positions according to these signals.
6. *Update the image on the PC:* The latest image captured by the video capture board is sent to the PC via the Ethernet for display.

This hardware implementation can run as an IPNC video surveillance system, which fits the active object during zoom operation. It can also run as the base of various applications, including active tracking [31,32], salient recognition [33], speed estimation [34] and automatic driving [35,36]. In this paper, we use a PC with a 2.6-GHz Intel Pentium Dual-Core CPU and 2 GB of memory for observation and control.

## 4. Experimental Results and Discussion

In this section, we provide a comparison of our FZT with the traditional zoom tracking approaches of GZT, AZT, RZT and PZT. The performance measures considered include tracking accuracy, tracking speed, storage space and training requirements. Tracking accuracy was measured in terms of mean offsets between the estimated and real trace curves for stationary and moving objects, respectively. Tracking speed was measured in terms of the total zoom operation time, which is dependent on the lens' motor type. Training and storage requirements were measured using the demand of determining the optimal model parameters. The parameters of the PI controller are also discussed in this section. Finally, the drawbacks of our method observed in the experiments are discussed.

All of the experiments were realised by the digital video surveillance system described in Section 3. Due to the four-subdivision mode, the zoom motor position, which was four times the normal pattern (190 to −970), ranged from 760 to −3,880. This mode improved the precision of our experiments. Furthermore, all of the experiments described in this section were under the zoom direction of wide-angle to tele-angle because the reverse sequence does not cause the one-to-many mapping problem when applied.

Moreover, because there are many independent parameters for our proposed system, we discuss how to obtain these values here. The seven main parameters in our algorithm are *K_P_*, *T_I_*, *T*, *fp*, *fra*, *bka* and *ps*. The proportional gain *K_P_*, integral time *T_I_* and sampling period *T* are three important parameters for the PI controller. We propose a combined tuning method for setting these three values in our experiments, as it is relatively difficult to obtain sufficient results using single tuning methods in complex surveillance environments. First, we use the Ziegler-Nichols [29] method to obtain the approximate values. Then, the manual tuning is performed for further optimisation according to the actual effect of the algorithm. The different revision effects acquired by the various *K_P_* and *T_I_* values in our experiments will be discussed in Section 4.3 as a reference for the stage of manual tuning. Because the *fp*, *fra*, *bka* and *ps* depend on different zoom lenses, image sensors, control circuits and application environments, it is difficult to find a common setting method for them. They should be regulated based on the hardware and software conditions and application environment, which can be obtained through several actual experiments in the user's specific working environment. We chose these values manually according to our digital surveillance platform and the scenes in our experiments. The feedback period *fp* controls the feedback frequency along the trace curve. A small *fp* value can increase the accuracy within a certain range through a frequent feedback procedure but causes increased time consumption and fluctuations on the trace curve. Thus, the *fp* value should achieve a balance between accuracy and user experience according to the specific application scene. Because user experience varies, this value setting mainly relies on actual tests and manual regulation. When there are many high-speed moving objects or objects with complex movement, such as in traffic or outdoor video surveillance, the value of *fp* should be relatively reduced. Otherwise, the *fp* should be set relatively high for indoor surveillance. The effect of *fp* in our experiment will be further discussed in Section 4.3 for advanced reference. The front area length *fra* and back area length *bka* are two auxiliary parameters that also affect the user experience by influencing the motor trace fluctuations. Their values are often set to 1/4 or 1/5 of the feedback period *fp*, depending on the user experience. The probe step length parameter *ps* controls the detection boundary of the algorithm, for which we have proposed an adaptive mechanism to determine this boundary described in Section 2.2.

### 4.1. Stationary Objects

The performance measures for tracking stationary object during zoom operation were collected from 600 distinct scenes under different lighting conditions and various object distances. This evaluation was performed for enlarged occasions in surveillance, which was described in Section 1.3. The object distances were set to 2, 3, 5, 10 and 20 m. For each distance, 120 samples were obtained from the GZT, AZT, RZT, PZT (S = 5), PZT (S = 20) and FZT models (20 samples for each method). Due to its higher accuracy in comparison to the RNN model [11], we chose the ARX model for PZT for all of our experiments. PZT(S = 5) indicates that the PZT model was only trained using five characteristic trace curves before use, whereas PZT (S = 20) was trained using 20 curves.

**Figure 13.**
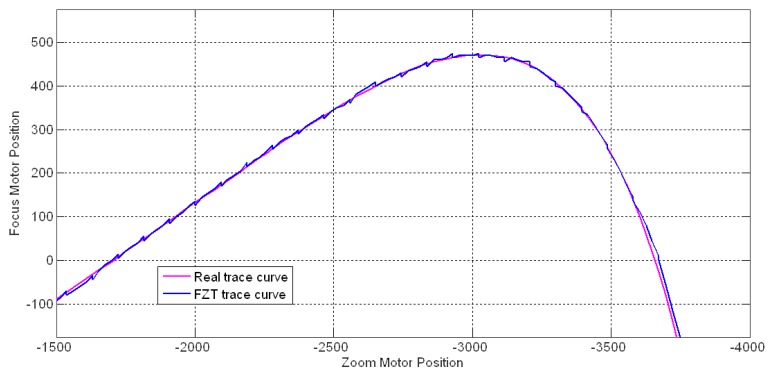
FZT trace curve for a 3 m stationary object.

Figure 13 shows an example of the trace curve for a 3 m stationary object acquired using our FZT method. In this case, the parameters were set as follows: *fp* = 96, *fra* = *bka* = 24, *K_P_* = 3, *T_I_* = 6, *T* = 1, and the adaptive probe step mechanism was applied to choose the *ps*. The real trace curve was obtained by running the global search auto-focusing function at each zoom motor position. The FZT trace curve was observed to tightly fit the real trace curve with several small fluctuations.

Table 1 summarises the overall tracking accuracy of the developed FZT compared with the existing GZT, AZT, RZT and PZT approaches. From this table, it can be observed that FZT exhibits better tracking accuracies than most of the traditional methods. However, FZT does not gain improvement in comparison with PZT trained by 20 trace curves due to its beneficial adaptability to the one-to-many mapping problem. However, if PZT has not been trained sufficiently, as shown in the PZT (S = 5) results, it may lose its advantages.

**Figure 14.**
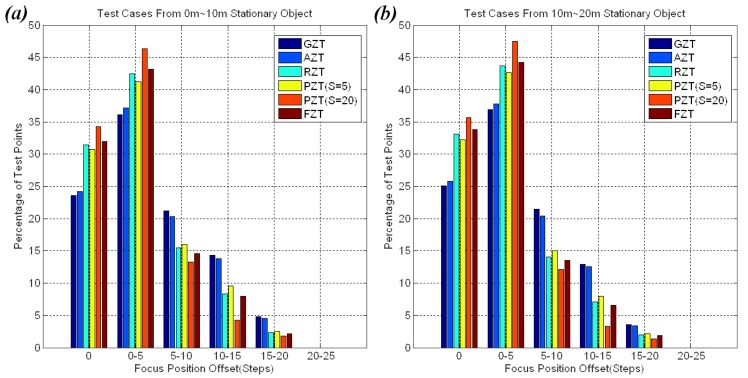
Offset distribution for stationary objects.

The distributions of offsets for all of the approaches in these experiments are shown in Figure 14. The cases are divided into two groups: 0 m to 10 m stationary objects and 10 m to 20 m stationary objects. The offsets of most points on the FZT trace curve were within five steps. The experiments also showed that there was a tolerant threshold of focus position offset for human vision. If the offset stays below the tolerant threshold, the user will not feel uncomfortable. This threshold is not a constant value but a variable that gradually increases from 10 to 30 steps when the zoom is changed from wide-angle to tele-angle in our system. Thus, the small fluctuations from probe steps on the FZT trace curve did not cause user discomfort.

**Figure 15.**
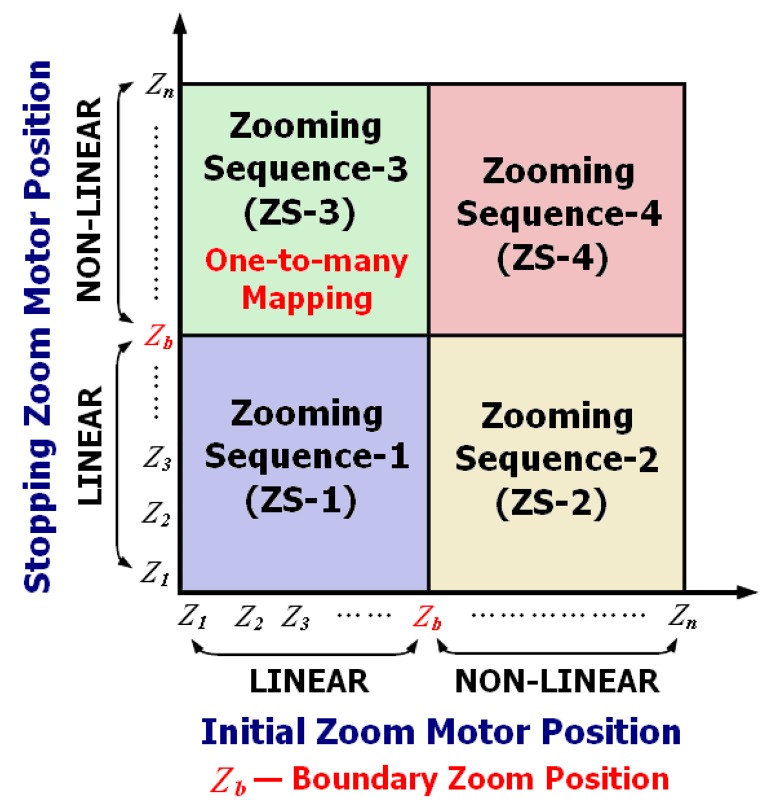
Different types of zooming sequences.

To compare the approaches for situations involving the one-to-many mapping problem, a further study was performed for the different zooming sequences shown in Figure 15. As indicated in this figure, the four different zooming sequences depend on the location of the initial and stopping zoom motor positions with respect to the boundary zoom position. Zooming Sequence-3 (ZS-3) incorporates the sequences that generate the one-to-many mapping problem because the zoom motor is moved from the linear region to the non-linear region on the trace curves.

A total of 400 experiments were performed to evaluate the performance for tracking an 8 m stationary object. For each zooming sequence, 100 samples were obtained using the GZT, AZT, RZT, PZT (S = 20) and FZT models (20 samples for each method). Table 2 provides the overall tracking accuracies for each sequence region. For stationary objects, PZT (S = 20) generated the least mean offset of 8.13 motor steps for ZS-3 compared to other approaches and worked better for the other three sequences as well. Furthermore, FZT exhibited a mean offset of 8.37 motor steps, which was more than that of PZT.

The FZT model was found to work better than most of the existing methods for tracking stationary objects with the exception of the PZT model with sufficient training. However, FZT does not require any specified training before tracking; thus, it is more suitable for use in complex environments in which the user is not able to acquire a sufficient amount of accurate training trace curves. It can also be applied to a video surveillance system with many different lens configurations in which the RZT or PZT models would have to be trained for every lens.

### 4.2. Moving and Switching Objects

**Figure 16.**
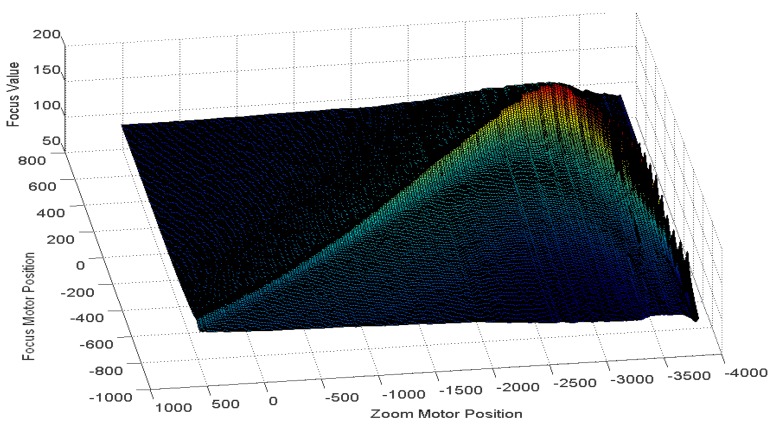
Focus value for per zoom position versus focus motor position.

Experiments were also performed to evaluate the robustness in tracking moving or switching objects. Figure 16 shows the focus values of an object moving from 6 m to 8 m. The focus values acquired by our equipment show an obvious real trace curve. Therefore, according to these values, the feedback mechanism can be run to revise the estimated trace curve.

**Figure 17.**
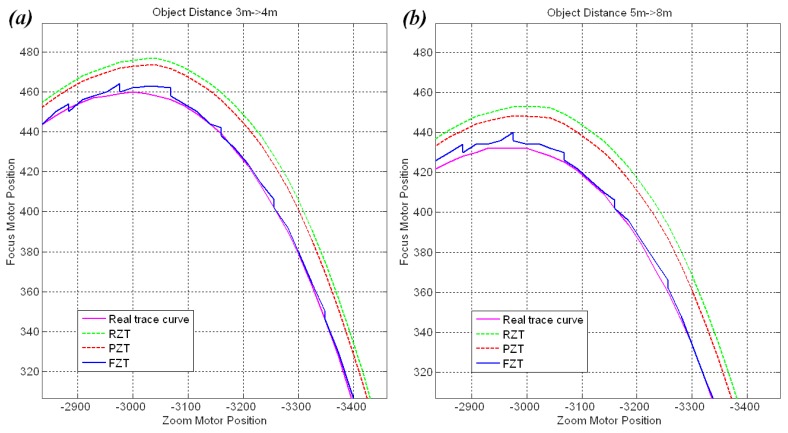
(**a**) Trace curves for an object moving from 3 m to 4 m; (**b**) Trace curves for an object moving from 5 m to 8 m.

Figure 17 shows the FZT trace curves for an object moving from 3 m to 4 m and an object moving from 5 m to 8 m compared with the RZT and PZT models. In these cases, the FZT parameters were set as follows: *fp* = 96, *fra* = *bka* = 24, *K_P_* = 1, *T_I_* = 8, *T* = 1, and the adaptive *ps* was used. The FZT trace curve was observed to be closer to the real trace curve than the RZT and PZT curves due to its real-time revision based on the feedback mechanism.

To further study the robustness in tracking moving or switching objects, we performed another 500 experiments for tracking objects moving from 2 m to 3 m, 5 m, 8 m, 10 m and 20 m. For each group of moving distance, 20 cases under different scenes for each tracking method were modelled. In these experiments, the FZT parameters were set as follows: *fp* = 96, *fra* = *bka* = 24, *K_P_* = 1, *T_I_* = 8, and *T* = 1.

Table 3 provides the results of the average tracking accuracy for these experiments. The FZT approach showed significant robustness, which was better than those of the other existing approaches. Furthermore, the mean offset of FZT grew slowly as the moving distance increased. The additional real-time estimate revision contributed to all of these effects.

Another 500 experiments under the similar parameter conditions were performed to validate the robustness for tracking switching objects in various scenes. We set two testing objects at 2 m and 3 m in the 2 m; 3 m group. When the main target switched from 2 m to 3 m, the FZT model exhibited the least mean offset of 8.41 motor steps in Table 4 compared with the other algorithms. Table 4 shows the overall accuracy results for this type of experiment.

Unlike moving object, switching object shows a transition in real trace curve because of the different object distances of switching targets in the scene. The focus value of image increases as motor positions approach the characteristic trace curve of new target. Thus, the estimated trace curve can be revised to the new object trace curve gradually towards the high focus value direction using real-time feedback mechanism of our FZT. The revision effect mainly focuses on a small range of the trace curve, in which the main object of image switches. Outside this range, FZT has little influence on the estimated trace curve. Experimental results show that FZT has better robustness compared with other existing methods on tracking switching object.

**Figure 18.**
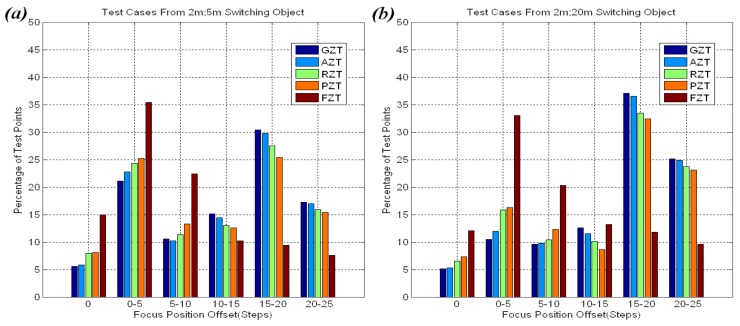
(**a**) Offset distribution for objects switching from 2 m to 5 m; (**b**) Offset distribution for objects switching from 2 m to 20 m.

Figure 18 shows the offset distributions for the 2 m; 5 m and 2 m; 20 m groups in the experiments. Most of the offsets on the FZT trace curve were within 10 steps, whereas more than 40% of the offsets on the other trace curves exceeded 15 steps. The large offset may cause users to be uncomfortable. Thus, FZT is the best choice for scenes that contain many moving or switching objects.

### 4.3. Control Parameters

**Figure 19.**
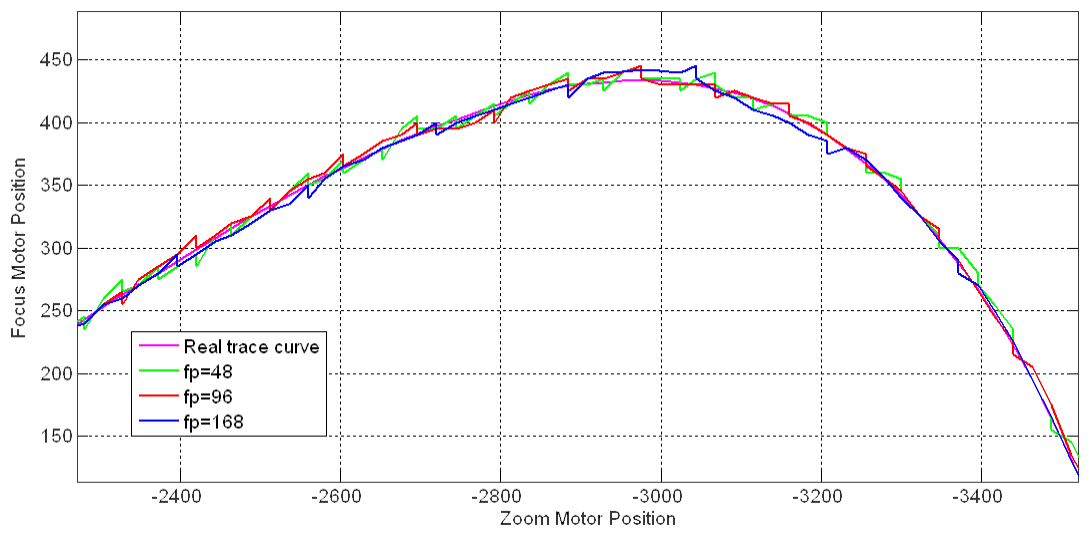
Trace curves for different FZT feedback periods.

The control parameter setting is an important problem in the applications of FZT applications. In this section we discuss the feedback period *fp*, proportional gain *K_P_* and integral time *T_I_*. The feedback period *fp* controls the feedback frequency along the estimated trace curve. Figure 19 shows the FZT trace curves for tracking an 8 m stationary object with different feedback periods under *fra* = *bka* = 24, *K_P_* = 3, *T_I_* = 6, and T = 1. A group of 20 experimental cases was performed for each *fp* value, and the average accuracies and time consumption are shown in Table 5. A small *fp* value caused the feedback procedure to occur frequently. In addition, it increased the accuracy within a certain range but caused a larger time consumption and more fluctuations along the trace curve. Moreover, the overly frequent revision might reduce the tracking accuracy at times due to the overshoot effect. Table 5 shows that *fp* = 96 was the suitable value for our device in this experiment due to the feedback procedure's high accuracy and relatively low time consumption.

After the discussion of *fp*, we consider the proportional gain *K_P_* for the PI controller. The parameter *K_P_* decides the revision magnitude. To show the magnitude in a clear manner, we use the feed response curves in which *fp* = *fa* and the motors are moved following the straight connection of probe points. For instance, if we want to produce the feedback response curve in Figure 9, the motors should be moved using the following sequence: *p_1_*, *p_2_*, *p′_1_*, *p′_2_*, *p″_1_*, *p″_2_*. This type of curve causes feedback operation throughout the time period and shows the revision distance ΔS directly through the amplitude of the curve.

**Figure 20.**
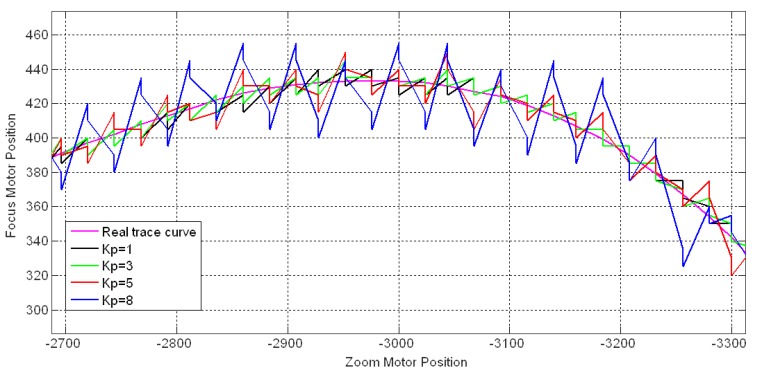
Feedback response curves for different proportional gains.

Figure 20 shows the feedback response curves for tracking the same 8 m stationary object with *T_I_* = 6, *T* = 1 and *K_P_* = 1, 3, 5, and 8. It was observed that the *K_P_* influenced the magnitude significantly. A high *K_P_* caused a large fluctuation on the response curve, which indicates strong adjustment on the estimated trace curve. In contrast, the small *K_P_* with a weak revision effect is not able to complement the error in time. Thus, the choice of *K_P_* should be based on the offset between the estimated and actual trace curves. For stationary objects, the *K_P_* can be set to a small value, whereas a larger *K_P_* is necessary for tracking moving or switching objects.

**Figure 21.**
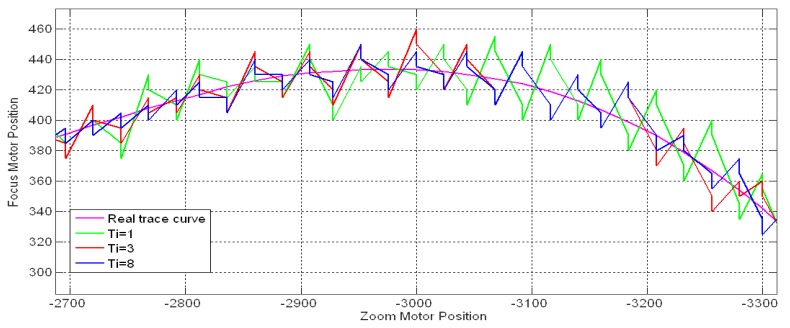
Feedback response curves for different integral time.

The integral term in the PI controller accumulates the past errors over time and adds them to the revision distance Δ*S* as a complementary effect. The parameter *T_I_* controls the speed of releasing the accumulated errors to the revision distance. Figure 21 illustrates the feedback response curves for tracking the same 8 m stationary object mentioned above with *K_P_* = 5, *T* = 1 and *T_I_* = 1, 3, and 8. As observed in Figure 21, a large *T_I_* value reduced the fluctuations of the response curve.

**Figure 22.**
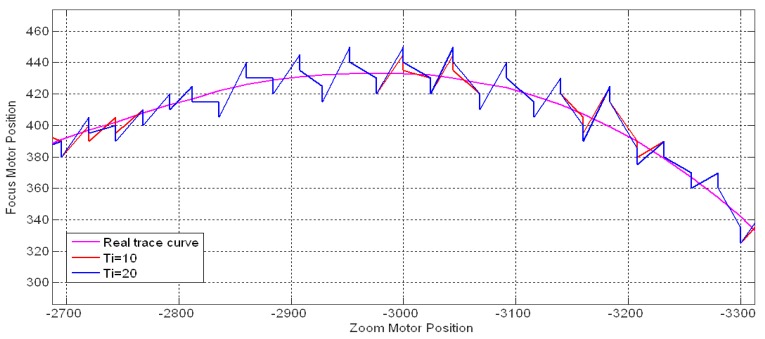
Complementary response curves for different integral time.

However, Figure 22 shows two additional cases with *T_I_* = 10 and 20 in which an excessively large *T_I_* could not achieve a sufficient feedback result because it reduced the role of the integral value. Thus, *T_I_* should be set properly according to the *K_P_* value, considering the revision effect and fluctuation.

### 4.4. Speed and Drawback

Because zoom tracking is a real-time application, tracking speed is also a key issue. Table 6 summarises the time consumption for the experiments of stationary, moving and switching targets. AZT took the largest amount of time due to its recalibration when crossing the boundary zoom position. FZT with *fp* = 96 achieved the second-highest time cost due to an additional 637 ms for feedback revision. Thus, FZT sacrifices speed in exchange for accuracy.

The comparison of other performance measures is summarised in Table 7. The following observations are made from this table. (1) GZT, AZT and FZT do not require any training procedures, while RZT and PZT require a minimum of 20 trace curves to generate an acceptable tracking result; (2) To revise the estimate, FZT requires some additional memory spaces, but this storage requirement does not grow as the number of discrete zoom motor position *N* increases. Thus, FZT limits the storage usage on the order of *N* similar to GZT, AZT and RZT, as opposed to PZT, which requires storage on the order of *N^2^*; (3) Unlike AZT, FZT does not cause discomfort when crossing the boundary zoom position. However, it causes users to be uncomfortable when the fluctuations on its trace curve are serious. Fortunately, this phenomenon occurs seldomly when we choose suitable parameters for the PI controller; (4) Based on the feedback, with respect to the moving or switching objects that often appear in video surveillance, FZT demonstrates robustness, while RZT and PZT have large offsets in these situations. Therefore, based on the above observations, FZT not only solves the one-to-many mapping problem but also improves the tracking robustness.

**Figure 23.**
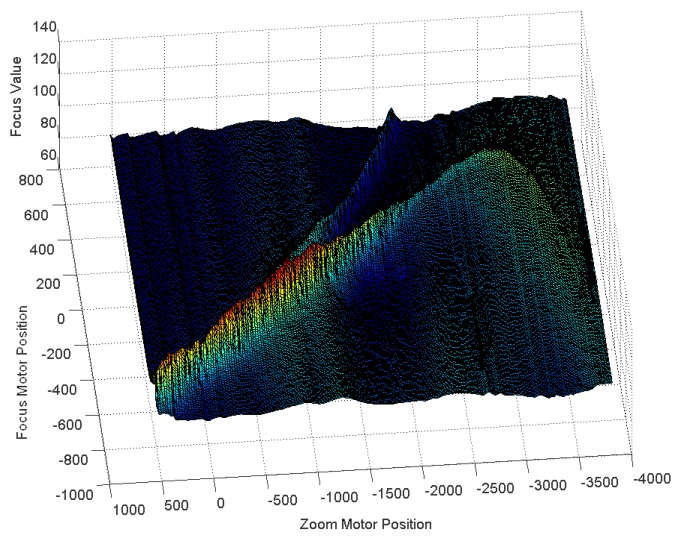
One example of FZT failure.

Finally, it is also worth mentioning that similar to GZT, AZT, RZT and PZT, our FZT method may also fail in several scenes in which there are two main targets at different distances due to an incorrect estimate acquired by auto-focusing at the beginning of the algorithms. Figure 23 shows one example of this failure. There are two peak lines in the figure that indicate the two main targets, whereas only one line is present in the normal case, as shown in Figure 16. The additional peak line will disturb the auto-focusing program and build an incorrect estimated trace curve due to its relatively high focus value. Due to this incorrect estimate at the beginning of the algorithm, FZT may fall into the local adjustment along the wrong curve.

It should also be noted that this drawback is not caused by the feedback mechanism but by the auto-focusing procedure. Thus, all of the existing zoom tracking approaches that use the auto-focusing program at the beginning of the algorithm have this drawback. The advanced auto-focusing technique concerning image content can further be used to cover this shortage.

## 5. Conclusions

In this paper, a robust feedback zoom tracking method has been introduced for digital video surveillance systems. This real-time method uses focus values and a PI loop-closed controller to revise the estimation of the trace curve. To assess performance, a real-time hardware implementation of the FZT algorithm along with commonly used methods was performed on an actual digital video platform. The extensive experiments under different lighting conditions for both stationary and moving objects revealed that the proposed feedback method generates better accuracies without pre-training compared to the commonly used approaches. Furthermore, the feedback mechanism may cause several fluctuations on the trace curve, but they typically stay within the tolerance level of a human being if the method parameters are properly chosen. Although it takes a little more time than traditional methods, the FZT method improves the robustness and adaptability of zoom tracking, particularly for moving or switching objects in video surveillance.

## Figures and Tables

**Table 1.** Tracking accuracy for stationary objects.

**Zoom tracking approach**	**Mean offset (motor steps) wide-angle > tele-angle**				

	**2 m**	**3 m**	**5 m**	**10 m**	**20 m**
GZT	7.68	7.36	7.13	6.85	6.59
AZT	7.45	7.16	6.95	6.69	6.46
RZT	5.92	5.64	5.46	5.22	5.04
PZT (S = 5)	6.14	5.87	5.68	5.45	5.23
PZT (S = 20)	5.15	4.89	4.73	4.52	4.34
FZT	5.79	5.53	5.34	5.10	4.91

**Table 2.** Tracking accuracy for each zooming sequence.

**Zoom tracking approach**	**Mean offset (motor steps) Object distance = 8 m**			

	**ZS-1**	**ZS-2**	**ZS-3**	**ZS-4**
GZT	3.19	3.18	9.07	6.22
AZT	3.34	3.32	7.79	6.56
RZT	2.86	2.95	8.68	5.65
PZT(S = 20)	2.49	2.33	8.13	4.61
FZT	2.63	2.57	8.37	5.13

**Table 3.** Tracking accuracy for moving objects.

**Zoom tracking approach**	**Mean offset (motor steps) wide-angle > tele-angle**				

	**2 m->3 m**	**2 m->5 m**	**2 m->8 m**	**2 m->10 m**	**2 m->20 m**
GZT	10.74	12.46	13.31	13.79	14.15
AZT	10.56	12.27	13.11	13.57	13.93
RZT	9.97	11.66	12.47	12.92	13.26
PZT(S = 20)	9.66	11.33	12.12	12.54	12.87
FZT	7.48	8.23	8.59	8.87	9.09

**Table 4.** Tracking accuracy for switching objects.

**Zoom tracking approach**	**Mean offset (motor steps) wide-angle > tele-angle**				

	**2 m; 3 m**	**2 m; 5 m**	**2 m; 8 m**	**2 m; 10 m**	**2 m; 20 m**
GZT	12.43	14.76	15.85	16.51	17.07
AZT	12.21	14.53	15.61	16.27	16.83
RZT	11.54	13.78	14.79	15.42	15.97
PZT(S = 20)	11.17	13.41	14.43	15.06	15.59
FZT	8.41	9.32	9.79	10.13	10.42

**Table 5.** Tracking accuracy and time consumption for different FZT feedback periods.

**Feedback period**	**Revision time**	**Total time**	**Mean offset**
*fp* = 48	1,274 ms	4,780 ms	5.21
*fp* = 72	849 ms	4,355 ms	5.14
*fp* = 96	637 ms	4,143 ms	5.18
*fp* = 120	510 ms	4,016 ms	5.24
*fp* = 144	425 ms	3,931 ms	5.37
*fp* = 168	364 ms	3,870 ms	5.59
*fp* = 192	318 ms	3,824 ms	6.04

**Table 6.** Time consumption of zoom tracking approaches.

**Zoom tracking approach**	**Auto-focus**	**Feedback revision**	**Trace curve estimation**	**Total time**
GZT	658 ms	0 ms	5 ms	3,506 ms
AZT	1,316 ms	0 ms	5 ms	4,164 ms
RZT	658 ms	0 ms	8 ms	3,509 ms
PZT	658 ms	0 ms	13 ms	3,514 ms
FZT	658 ms	637 ms	5 ms	4,143 ms

**Table 7.** Performance comparison of zoom tracking approaches.

**Performance measures**	**Zoom tracking approach**				

	**GZT**	**AZT**	**RZT**	**PZT**	**FZT**
Requires training	No	No	Yes	Yes	No
Number of trace curves for training	0	0	20	20	0
Storage usage (*N* zoom positions)	3N	3N	3N	3N^2^	3N
Causes user discomfort during zooming	No	Boundary	No	No	Seldom
Robustness for moving or switching objects	No	No	No	No	Yes

## References

[1] Haritaoglu I., Harwood D., Davis L.S. (2000). W-4: Real-time surveillance of people and their activities. IEEE Trans. Pattern Anal. Mach. Intell..

[2] Foresti G.L., Micheloni C., Piciarelli C., Snidaro L. (2009). Visual sensor technology for advanced surveillance systems: Historical view, technological aspects and research activities in Italy. Sensors.

[3] Chen Y.-L., Chiang H.-H., Chiang C.-Y., Liu C.-M., Yuan S.-M., Wang J.-H. (2012). A vision-based driver nighttime assistance and surveillance system based on intelligent image sensing techniques and a heterogamous dual-core embedded system architecture. Sensors.

[4] Tordoff B.J., Murray D.W. (2007). A method of reactive zoom control from uncertainty in tracking. Comput. Vis. Image Underst..

[5] Fayman J.A., Sudarsky O., Rivlin E., Rudzsky M. (2001). Zoom tracking and its applications. Mach. Vis. Appl..

[6] Cheng H.Y., Hsu S.H. (2011). Intelligent highway traffic surveillance with self-diagnosis abilities. IEEE Trans. Intell. Transp. Syst..

[7] Lee Y.S., Chung W.Y. (2012). Visual sensor based abnormal event detection with moving shadow removal in home healthcare applications. Sensors.

[8] Kumar P., Ranganath S., Huang W., Sengupta K. (2005). Framework for real-time behavior interpretation from traffic video. Intell. Transp. Syst. IEEE Trans..

[9] Gamadia M., Peddigari V., Kehtarnavaz N., Lee S.-Y., Cook G. Real-time implementation of autofocus on the TI DSC processor.

[10] Weerasinghe C., Nilsson M., Lichman S., Kharitonenko I. (2004). Digital zoom camera with image sharpening and noise suppression. IEEE Trans. Consum. Electron..

[11] Peddigari V., Kehtarnavaz N. (2007). Real-time predictive zoom tracking for digital still cameras. J. Real-Time Image Process.

[12] Hoad P., Illingworth J. Automatic Control of Camera Pan, Zoom and Focus for Improving Object Recognition.

[13] Kim Y., Lee J.S., Morales A.W., Ko S.J. (2002). A video camera system with enhanced zoom tracking and auto white balance. IEEE Trans. Consum. Electron..

[14] Peddigari V.R., Kehtarnavaz N., Lee S.-Y., Cook G. Real-time implementation of zoom tracking on TI DM processor.

[15] June-Sok L., Sung-Jea K., Yoon K., Morales A. A video camera system with adaptive zoom tracking.

[16] Peddigari V., Kehtarnavaz N. (2005). A relational approach to zoom tracking for digital still cameras. IEEE Trans. Consum. Electron..

[17] Wang D., Ding F. (2010). Input-output data filtering based recursive least squares identification for cararma systems. Digit. Signal Process..

[18] Kamijo K., Tanigawa T. Stock price pattern recognition-a recurrent neural network approach.

[19] Sánchez J., Benet G., Simó J.E. (2012). Video sensor architecture for surveillance applications. Sensors.

[20] Hu H.G., Xu L.H., Wei R.H., Zhu B.K. (2011). Multi-objective control optimization for greenhouse environment using evolutionary algorithms. Sensors.

[21] Jimenez-Fernandez A., Jimenez-Moreno G., Linares-Barranco A., Dominguez-Morales M.J., Paz-Vicente R., Civit-Balcells A. (2012). A neuro-inspired spike-based PID motor controller for multi-motor robots with low cost fpgas. Sensors.

[22] Yu Z.P., Wang J.D., Huang B.A., Bi Z.F. (2011). Performance assessment of PID control loops subject to setpoint changes. J. Process. Control.

[23] Bennett S. (1993). A History of Control Engineering, 1930–1955.

[24] Kehtarnavaz N., Oh H.J. (2003). Development and real-time implementation of a rule-based auto-focus algorithm. Real-Time Imaging.

[25] Peddigari V., Gamadia M., Kehtarnavaz N. (2005). Real-time implementation issues in passive automatic focusing for digital still cameras. J. Imaging Sci. Technol..

[26] Kuo C.F.J., Chiu C.H. (2011). Improved auto-focus search algorithms for cmos image-sensing module. J. Inf. Sci. Eng..

[27] Burge J., Geisler W.S. (2011). Optimal defocus estimation in individual natural images. Proc. Natl. Acad. Sci. USA.

[28] Lee J.-Y., Wang Y.-H., Lai L.-J., Lin Y.-J., Chang Y.-H. (2011). Development of an auto-focus system based on the moiré method. Measurement.

[29] Åström K.J., Hägglund T. (2004). Revisiting the ziegler-nichols step response method for PID control. J. Process Control.

[30] Ho W.K., Hang C.C., Zhou J.H. (1995). Performance and gain and phase margins of well-known PI tuning formulas. Control Syst. Technol. IEEE Trans..

[31] Huang J.W., Li Z.N. (2011). Automatic detection of object of interest and tracking in active video. J. Signal Process. Syst. Signal Image Video Technol..

[32] Kumar P., Dick A., Sheng T.S. Real Time Target Tracking with Pan Tilt Zoom Camera.

[33] Kwak S., Ko B., Byun H. (2007). Salient human detection for robot vision. Pattern Anal. Appl..

[34] Doǧan S., Temiz M.S., Külür S. (2010). Real time speed estimation of moving vehicles from side view images from an uncalibrated video camera. Sensors.

[35] Lin C.-C., Wang M.-S. (2012). A vision based top-view transformation model for a vehicle parking assistant. Sensors.

[36] Garcia-Garrido M.A., Ocana M., Llorca D.F., Arroyo E., Pozuelo J., Gavilan M. (2012). Complete vision-based traffic sign recognition supported by an i2v communication system. Sensors.

